# Striatal GDNF Production Is Independent to Circulating Estradiol Level Despite Pan-Neuronal Activation in the Female Mouse

**DOI:** 10.1371/journal.pone.0164391

**Published:** 2016-10-14

**Authors:** Daniel Enterría-Morales, Ivette López-López, José López-Barneo, Xavier d’Anglemont de Tassigny

**Affiliations:** 1 Instituto de Biomedicina de Sevilla (IBIS), Departamento de Fisiología Médica y Biofísica, Hospital Universitario Virgen del Rocío/ CSIC/Universidad de Sevilla, Sevilla, Spain; 2 Centro de Investigación Biomédica en Red sobre Enfermedades Neurodegenerativas (CIBERNED), Madrid, Spain; Hudson Institute, AUSTRALIA

## Abstract

Gender difference in Parkinson’s disease (PD) suggests that female sex steroids may promote dopaminergic neuron survival and protect them from degeneration. The glial cell line-derived neurotrophic factor (GDNF) is believed to be dopaminotrophic; thus it is considered as a potential therapeutic target in PD. Additionally, GDNF is endogenously synthetized in the caudate/putamen of humans and striatum in rodents. A neuroprotective role of estrogens on the nigrostriatal pathway via the stimulation of GDNF has been proposed. Since the GDNF-producing parvalbumin (Parv) interneurons express the estrogen receptor alpha in the mouse striatum, we sought to determine whether ectopic estrogenic compound modulates the GDNF synthesis in mice. Using an ovariectomized-estradiol (E_2_) replacement regimen, which reliably generates a rise of plasma estradiol, we assessed the effects of different levels of E_2_ on the activation of striatal neuronal populations, and GDNF production. A strong correlation was found between plasma E_2_ and the expression of the immediate early gene cFos in the striatum, as well as in other cortical regions. However, moderate and high E_2_ treatments failed to induce any striatal GDNF mRNA and protein synthesis. High E_2_ only stimulates cFos induction in a low percentage of striatal Parv neurons whereas the majority of cFos-positive cells are medium spiny neurons. Activation of these projecting neurons by E_2_ suggests a role of circulating sex steroids in the modulation of striatal neural pathways.

## Introduction

Estrogen is an important hormone signal that regulates multiple tissues and functions in the body. Besides modulating the development and regulation of the female reproductive system and secondary sex characteristics, estrogen has a neurotrophic and neuroprotective role in the brain [1,2]. For instance, gender difference exists in the prevalence of patients with Parkinson’s disease (PD), a degenerative disorder mainly characterized by the progressive loss of mesostriatal dopamine (DA) neurons, particularly in the substantia nigra pars compacta (SNpc) [3]. The male population is generally more susceptible to develop PD symptoms such as tremor, dyskinesia and rigidity [4–6]. Likewise, menopause and steroid treatments affect the early symptoms: they usually appear sooner in early-menopausal women while estrogen-based therapies delay PD onset [7].

Multiple studies in rodent and primate PD models showing pro-dopaminergic actions of estrogen reinforced the aforementioned clinical observations [8]. A greater susceptibility of the nigrostriatal dopaminergic pathway to 6-hydroxydopamine (6-OHDA) is observed in male rats [9]. Moreover, estrogenic supplementation usually prevents degeneration of DA neurons after neurotoxin treatment with 1-methyl-4-phenyl-1,2,3,6-tetrahydropyridine (MPTP) in mouse and 6-OHDA in rat models [10–12]. The question regarding which estrogen receptor (ER) mediates the estrogen signaling has been well studied. Immunolocalization experiments indicate that nuclear ERβ is hardly expressed in the mouse striatum in comparison to ERα [13]. Moreover, ER knock-out mice, particularly those lacking ERα, are more susceptible to MPTP toxicity [14,15]. Likewise, specific ERα agonists induce protection against MPTP-induced striatal DA depletion, while ERβ agonists have no such efficiency [11,16]. In opposition to the aforementioned findings, absence of neuroprotection by estrogen, or even antidopaminergic effects, have also been described and discussed in PD animal models [12]. For instance, estradiol replacement in ovariectomized (ovx) rats failed to prevent the OHDA-induced DA loss [17].

Since there is no therapy that prevents the loss of DA neurons [18], the neuroprotective action of estrogen is intriguing and understanding its mode of action is important. Indeed, the mechanisms by which estrogen protects the nigrostriatal DA pathway remain relatively unknown. However, it is established that estrogen signaling induces anti-apoptotic genes and growth factors, and inhibits the synthesis of pro-apoptotic genes and pro-inflammatory factors [1]. Among the wealth of potent pro-dopaminergic molecules, glial cell line-derived neurotrophic factor (GDNF) is a potential candidate for mediating the estrogen neurotrophic action. The neurotrophic role of GDNF on catecholaminergic neurons is well recognized [19], however clinical trials assessing exogenous GDNF delivery on PD patients have been unsuccessful thus far. Nonetheless, GDNF is endogenously produced in the brain, particularly in the striatum, and retrogradely transported to the SNpc by the mesencephalic DA neurons of the nigrostriatal pathway [20]. While GDNF deletion effects are controversial [21–23], the results of GDNF elevation are indisputably positive [19]. The most recent and significant data supporting the neuroprotective role of endogenous GDNF on mesencephalic DA neurons were obtained from *Gdnf* hypermorphic mice (*Gdnf* ^*hyper*^) that express twice the normal GDNF levels in the striatum. The subsequent endogenous GDNF elevation increases the number of DA neurons during development, and is retained in adulthood [24]. Importantly, a two-fold elevation in endogenous GDNF levels protects the *Gdnf* ^*hyper*^ mice from lactacystin toxicity in a PD model, without the side effects associated to ectopic GDNF applications. Thus, recent advances gained from transgenic models suggest that striatal GDNF plays a key role in the maintenance of the nigrostriatal DA pathway and constitutes a potential target to fight against the progressive motor decline associated to PD.

Two recent studies have linked estrogens to GDNF, indicating that the neurotrophic factor synthesis may be modulated by estradiol. Mini-osmotic pumps delivering E_2_ promoted estrogenic neuroprotection against 6-OHDA-induced nigrostriatal lesion in male rats. This neuroprotection effect of E_2_ was associated to a fair increase of GDNF protein content in both the substantia nigra and the striatum [25]. Interestingly, in mesencephalic neuron-glia co-cultures, blockade of the GDNF up-regulation by RNA interference or neutralizing antibodies prevents the E_2_ neuroprotective effect [25]. Additionally, it has been suggested that the G protein-coupled estrogen receptor (GPER) may be involved in the E_2_-induced GDNF synthesis in primary cultures of embryonic midbrain [26]. These recent advances on the role of estrogen as a neuroprotective factor, and its stimulating action on GDNF, unveil new therapeutic alternatives in PD research. However, in an effort to further understand the interaction of E_2_ with GDNF, and its putative relevance in PD therapy, it is worth investigating the effect of estrogen in different models. In this context, we chose to work with a model of ovariectomized (ovx) female mouse, whose depletion of endogenous gonadal estrogen was replaced by 17β-estradiol (E_2_), the most biologically prevalent and active estrogenic compound.

We report here the effect of circulating E_2_ on the neuronal activation and GDNF levels in the mouse striatum. First, we set out to determine the expression of ERα in striatal interneurons and projection neurons. Secondly, we evaluated the effect of circulating E_2_ on the striatal GDNF synthesis at both gene expression and protein content. Finally, to further understand the action of high circulating E_2_ in the striatum, we examined the expression pattern of cFos, an indirect marker of neuronal activity, in the main populations of striatal neurons.

## Materials and Methods

### Animals

Experiments were undertaken on adult (2 months of age) wild-type C57BL/6J mice supplied by Charles River (France). All mice were housed under a 12:12 h lighting schedule (lights on at 7:00 a.m.) with *ad libitum* access to food and water. Heterozygous male and female mice (4 months of age) for GDNF (Gdnf^+/-^) were produced in our animal facility and genotyping was performed as described in Pascual et al. (2008) [21]. All experimental protocols were approved by the Ethical committee of the Virgen Macarena and Virgen del Rocío hospitals (Seville, Spain) under the project licence no. 27-05-15-255 delivered by the Council of Andalusia.

### Reagents

We used the following primary antibodies as follows: anti-Akt (9272, Cell Signaling) at 1:1000; anti-phospho(Ser473)-Akt (9271, Cell Signaling) at 1:1000; anti-cFos (PC38, Calbiochem) at 1:10000; anti-Darpp32 (AB10518, Millipore) at 1:2500; anti-ERα (06–935, Millipore) at 1:500; anti-Parvalbumin (PVG214, Swant) at 1:5000. In the immunostaining procedure, we used secondary antibodies anti-rabbit IgG and anti-goat IgG conjugated with Alexa Fluor 488 or Alexa Fluor 568 at 1:1000 (Invitrogen), AffiniPure Fab Fragment Goat Anti-rabbit IgG (H+L) (111-007-003, Jackson Laboratories). In the Western blot protocol, we used HRP-conjugated secondary antibodies anti-rabbit IgG (31460, Thermo Scientific) and anti-mouse IgG (NA931V, GE Healthcare) at 1:10000. The specificity of each antibody used in this study has been extensively tested by other groups and showed specific and expected staining in our hands.

For quantitative PCR, we used the following TaqMan probes (from Thermofisher): *Gdnf1-2* (Gdnf exons 1 to 2), Mm00599849_m1; *Pvalb* (parvalbumin), Mm00443100_m1; *Actb* (βActin), Mm00443100_m1; *Gapdh*, Mm99999915_g1; *Hmbs*, Mm00660262_g1.

17β-estradiol (E8875, Sigma) was used to make up the SILASTIC implants, and β-estradiol 3-benzoate (E-8515, Sigma) for subcutaneous injection.

### Ovariectomy / estradiol replacement

To remove the source of circulating gonadal hormones mice were treated as reported previously [27]. In brief, female mice were anesthetized with ketamine / xylasine dosage according to the Cold Spring Harbor Protocols 2006, bilaterally ovariectomized, and given subcutaneous SILASTIC implants containing 17β-estradiol (E_2_; 1 μg / 20 g of body weight) according to Bronson (1981) [28]. The SILASTIC capsules are made by dissolving crystalline E_2_ with ethanol, and mixing it with SILASTIC medical adhesive, which is then injected into 1 mm (internal diameter) x 2.125 mm (external diameter) SILASTIC tubing (Dow Corning, USA). Once dried, the tubing is cut to size depending on the weight of the mouse such that a 20 g mouse receives a 1 cm length of tubing (1 μg of E_2_). Mice were treated with 2.5 mg / kg b.w. Meloxicam i.p. post-surgery, and paracetamol 2 g / l in drinking water during the 10 days following surgery. Three weeks after ovx, mice received an s.c. injection of estradiol benzoate (2 μg / 20 g of b.w.) or vehicle (corn oil) at 12:00 p.m.

Four treatment groups were constituted: i/ group O received SILASTIC implants that contain SILASTIC medical adhesive free of E_2_ and vehicle 3 weeks later, ii/ group Oi received E_2_ SILASTIC + vehicle, iii/ group E1 received E_2_ SILASTIC + one s.c. injection of E_2_, iiii/ group E5 received E_2_ SILASTIC + five s.c. injection of E_2_ during 5 consecutive days.

Mice in groups O, Oi and E1 were sacrificed 24h after s.c. injection of E_2_ or vehicle. Mice in group E5 were sacrificed 4–5 hours after the last s.c. injection of E_2_.

### Estradiol assay

17-β-estradiol was measured using the Estradiol EIA Kit (582251, Cayman Chemical), following the manufacturer’s recommended protocol, with an intra-assay sensitivity of 10 pg/ml. 50 μL of plasma were assayed in duplicate for each mouse. 17-β-estradiol concentration was calculated using the equation obtained from the standard curve plot, and expressed as pg/ml to perform comparative analysis.

### Protein extraction

Protein extracts were prepared in 400μl of lysis buffer / striatum and 600 μl / cortex (pH 7.4, 25 mM Tris, 50mM β-glycerophosphate, 1.5 mM EGTA, 0.5 mM EDTA, 1 mM sodium pyrophosphate, 1 mM sodium orthovanadate, 100 μg/ml PMSF, 1% v/v protease inhibitors cocktail (Sigma, P8350), 1% v/v phosphatase inhibitors cocktail (Sigma, P0044) and 1% Triton X-100) by trituration of the fragments through 22 and 25 gauge needles in succession. Homogenates were incubated on ice for 15 min, followed by centrifugation at 16,000 x *g* for 15 min. The supernatants were removed and frozen. Protein content was determined using the Bradford method (Bio-Rad, Hercules, CA).

### Western blot

15 μg of protein (from striata samples) were run on a 10% SDS-PAGE gel, using Mini-Protean TGX Stain-Free Precast Gels (BioRad), and transferred to PVDF membrane (Immobilon^®^-P, Millipore). Membrane was blocked in PBS 0.05%, Tween20, 5% BSA for 1 hr at room temperature (RT) and then incubated overnight at 4°C with primary antibody prepared in the same blocking solution. After washing steps, membrane was incubated with horseradish peroxidase-conjugated secondary antibody 1 hr at RT. Blots were revealed with Clarity^TM^ Western ECL substrate (BioRad) and scanned by a luminescent image analyzer (ImageQuant LAS 4000mini, GE Healthcare). Specific bands intensity was quantified as optical density using ImageJ software. The ratio pAkt / Akt was calculated to quantify the Akt phosphorylation level in each group.

### Real time quantitative PCR

Total RNAs were isolated with Trizol reagent (Life Technologies) from striatum and cortex, according to the manufacturer’s directions. 500 ng of total RNA was copied to cDNA using QuantiTect Reverse Transcription Kit (Qiagen) in a final volume of 20 μl. Real-time quantitative PCR reactions were performed in a 7500 Fast Real Time PCR System (Life Technologies). PCR reactions were performed in duplicates in a total volume of 20 μl containing 5 μl of cDNA solution and 1 μl of Taqman probe of the specific gene (Life Technologies). Housekeeping genes expression, such as *Actb*, *Gapdh* or *Hmbs*, were also estimated in each sample to normalize the amount of total RNA input in order to perform relative quantifications.

### GDNF protein assay

Striatal GDNF protein content was estimated using a commercial enzyme-linked immunosorbant assay (ELISA) kit (GDNF Emax Immunoassay System; Promega). 80 μg of protein were used in the assay. ELISA was performed following the manufacturer’s protocol with minor modifications. Protein extracts of cortex from the same mice were used as negative controls to set background levels since *Gdnf* is not detected in the mouse cortex (see S1 File). GDNF protein value calculated from cortex samples was subtracted to each individual measurement. The final value expressed as pg/mg total protein was used to perform comparative analysis.

### Experiment 1

Six mice per group were sacrificed by overdose injection of thiobarbital 0.6 g / kg b.w. (B. Braun, Jaén, Spain). A 0.5 ml blood sample was taken from the inferior vena cava, mixed with 5 μl EDTA and centrifuged at 10,000 x g for 10 min at 4°C. The plasma supernatant samples were collected and stored at -20°C until assayed. Brain was rapidly removed, washed three times in ice-cold sterile PBS prepared with DEPC H_2_O, two cortexes and two striata per brain were dissected out on ice-cold sterile plastic plate. Each fragment was placed in a microcentrifuge tube, snap frozen in liquid nitrogen, and stored at -80°C. Cortex tissue dissection mainly includes the somatomotor, somatosensory and auditory areas of the isocortex. Striatum fragment consisted of the whole dorsal striatum.

### Experiment 2

3 to 4 female mice per experimental group were used for immunohistochemistry experiments. Mice were deeply anesthetized with thiobarbital as described above and intracardially perfused with phosphate-buffered saline 0.1 M (PBS) followed by 4% paraformaldehyde in PBS pH 7.4. After a 1 hr post fixation step, brains were washed in PBS, cryoprotected in PBS with 30% sucrose, embedded in Tissue-Tek^®^ O.C.T. Compound (Sakura^®^, Finetek), and frozen on dry ice. Using a cryostat (CM 1950, Leica, Germany), 30 or 50 μm coronal floating sections were obtained and stored in an antifreeze solution (0,9% w/v NaCl, 30% w/v sucrose, 1% w/v Polyvinylpyrrolidone (9003-39-8, Sigma), 30% v/v Ethylene glycol in PBS) at −20°C until use. Primary and secondary antibodies were prepared in PBS with 0.1% Triton X-100, 10% fetal bovine serum and 1 mg/ml bovine serum albumin. Sections were incubated with primary antibodies (Parv, Darpp32, cFos, ERα) overnight at 4°C, and then incubated with secondary anti-IgG antibodies conjugated with Alexa Fluor^®^ 488 or 568 for 2 hrs at room temperature. When a double staining with two rabbit serum antibodies was required, an additional 2 hrs blocking step using unconjugated goat anti-rabbit IgG at 1:1000 was done between the first and second incubations with primary / secondary antibodies. Nuclei were detected by 4’,6’-diamidino-2-phenylindole (Dapi, D9542, Sigma). Fluorescence images were obtained with a BX61 microscope equipped with a DP70 camera (Olympus). We used 5 successive brain sections (150μm between each section) per mouse to take images of 10 regions of interests (ROIs) of the striatum and 5 ROIs of the motor cortex. Photographs of cFos-immunostained slices were analyzed with custom macro automation quantification by ImageJ software to count the number of cFos^+^ cells. Using the same ROIs, we manually quantified the number of double positive cells in the striatum. Values were reported as cells (PV+ cFos+, Darpp32+ cFos+, PV+ ERα+, Darpp32+ ERα+) per mm^3^, from the average of the data obtained from all ROIs, the ROI area (0.395 mm^2^) and the sample thickness (30 or 50 μm).

### Experiment 3

Three male and three female mice were used for immunohistochemical mapping of ERα, with the same immunostaining methods as described above.

### Statistics

We used Graphpad—Prism 6 software to perform statistical comparisons and to calculate Pearson’s correlation. Before statistical analysis, percentages were subjected to arc-sine transformation to convert them from a binomial to a normal distribution. Comparison between two groups was subjected to an unpaired Student’s *t*-test. One-way analysis of variance (ANOVA), followed by Tukey post hoc multiple comparisons test, was used to draw comparisons between three or more groups. The level of significance was set at *p <* 0.05.

## Results

**Fig 1:**
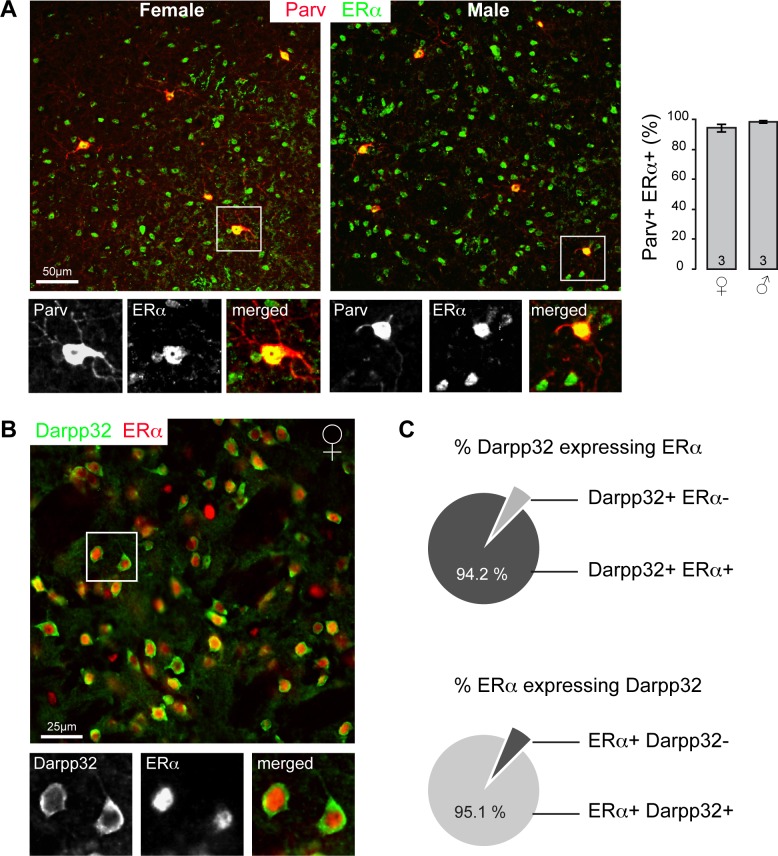
Estrogen receptor alpha (ERα) expression in the mouse striatum. (A) Photomicrographs show parvalbumin (Parv) (red) and ERα+ (green) staining in male and female coronal sections. Vertical bargraphs on the right indicate the mean percentage ± SEM of the Parv+ cells that are ERα+ (n = 3). (B) microphotograph illustrating ERα (red) expression by Darpp32+ medium spiny neurons (green) in a female brain. Note that ERα is almost exclusively expressed in Darpp32+ cells. (C) Quantification of the Darpp32 and ERα colocalization shows that 94.2 ± 1.9% of the Darpp32+ neurons are immuno-positive for ERα; and 95.1 ± 0.8% of striatal ERα is expressed in Darpp32+ neurons in male and female mice (n = 4, from 2 male and 2 female striata).

Estrogen receptor alpha (ERα) is the ER subtype involved in the E_2_-mediated neuroprotection [14], and is widely expressed in the mouse brain [13] (Fig A in S1 File). Parvalbumin (Parv) interneurons account for the majority of the GDNF synthesizing cells in the striatum [24,29]. Dual-labeling immunocytochemistry showed the presence of ERα in 94.1 ± 2.5% and 98.4 ± 0.9% of Parv neurons throughout the striatum of female (n = 3) and male (n = 3) respectively (Fig 1A). These data indicate that Parv neurons are equipped with the appropriate estrogen receptor to take part in the neuroprotection mechanism promoted by circulating E_2_. In addition, we observed the expression of ERα in striatal medium spiny neuron (MSN) by dual staining with dopamine- and cAMP-regulated neuronal phosphoprotein (Darpp32) (Fig 1B), a protein that is specifically expressed in neurons that also express DA receptors [30]. A thorough inspection of male and female striata revealed that nearly every single MSN expresses ERα (94.2 ± 1.9%), as quantified in brains from two female and two male (n = 4) (Fig 1C). Likewise, MSN count for the large majority of the ERα positive cells in the striatum (95.1 ± 0.8%) (Fig 1C), whereas the remaining ERα-expressing cells are presumably interneurons, including Parv neurons (Fig A in S1 File).

**Fig 2:**
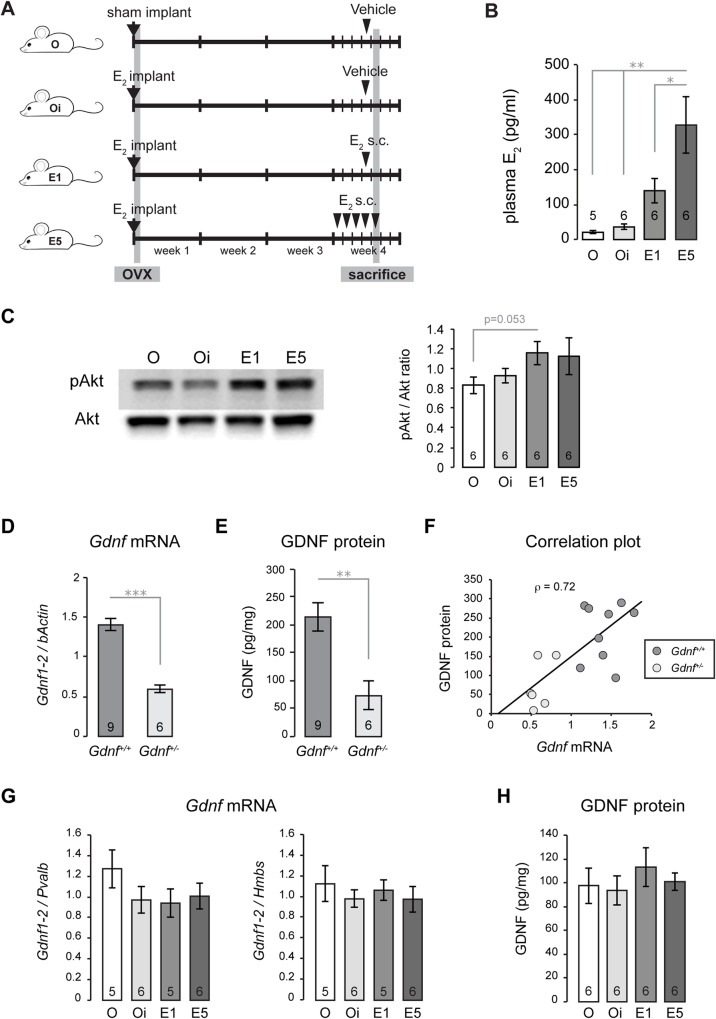
Absence of GDNF modulation by estradiol replacement in ovariectomized female mice. (A) Mouse experimental model. Ovariectomized (ovx) animals were separated in four groups and received either sham implant (O) or E_2_ implant (Oi, E1 and E5) at surgery time. Three weeks later, mice received Vehicle (O and Oi), a single s.c. E_2_ injection (E1), or five E_2_ injections over 5 days (E5). Animals were sacrificed 24 hours after s.c. injection (O, Oi, E1) or 4–5 hours after the last injection (E5). (B) Vertical bar graph showing the mean ± SEM of plasma E_2_ in O, Oi, E1 and E5 mice. This shows that the estrogen replacement protocol induced an increase in circulating E_2_ in ovx mice. (C) Western blot analysis of phosphorylated Akt (pAkt) showing a representative blot (left), and pAkt / Akt ratio is shown with a vertical bar graph (right). The greatest difference is found between O and E1 (p = 0.053). (D) *Gdnf* mRNA expression, reported as *Gdnf1-2* relative to *bActin*, in the striatum of wild-type (*Gdnf*^*+/+*^) and heterozygous mice (*Gdnf*^*+/-*^). (E) GDNF protein content expressed as pg / mg total protein in *Gdnf*^*+/+*^ and *Gdnf*^*+/-*^ striatum. (F) Scatter plot diagram showing the positive correlation (ρ = 0.72, p = 0.002) between *Gdnf* mRNA (shown in D) and GDNF protein levels (shown in E) in *Gdnf*^*+/+*^ and *Gdnf*^*+/-*^ striata. This data shows that GDNF protein concentration measured by ELISA is consistent with *Gdnf* gene expression level. (G) QPCR analysis of *Gdnf* expression measured by *Gdnf1-2* relative to *Parv* mRNA (left graph) or *Hmbs* mRNA (right). (H) ELISA analysis of GDNF protein levels showing no difference between the experimental groups. All data are presented as the mean ± SEM. *, p < 0.05; **, p < 0.01; ***, p < 0.001. N is indicated at the bottom of each bar.

To study the effect of exogenous E_2_ administration, we divided the animals in four experimental groups labeled O, Oi, E1 and E5 as follows: ovariectomized without any exogenous E_2_ (O), ovariectomized with basal E_2_ continuously released from a SILASTIC implant (Oi), ovariectomized with E_2_ SILASTIC implant + one E_2_ injection (E1), and ovariectomized with E_2_ SILASTIC implant + five E_2_ injections over 5 days (E5) as depicted in Fig 2A. E_2_ treated mice exhibited an E_2_-dependant significant increase of blood circulating E_2_ measured in plasma at sacrifice (Fig 2B). O, 20.8 ± 3.6 pg/ml (n = 5); Oi, 36.0 ± 7.9 pg/ml (n = 6); E1, 138.8 ± 34.4 pg/ml (n = 6); E5, 327.5 ± 80.6 pg/ml (n = 6). Vaginal cytology evaluation at sacrifice indicated that the hormonal replacement was efficient. O mice only presented leucocytes; Oi mice had a mix of leucocytes and large nucleated epithelial cells, whereas mice from E1 and E5 groups presented only cornified cells (Fig B in S1 File), which indicates a high steroidal impregnation as it is normally observed in the estrus stage of the estrous cycle in rodents [31]. Interestingly, *Esr1* mRNA expression (gene coding for ERα) did not change upon the increase of blood E_2_ levels (Fig A in S1 File*)*. Estrogen-mediated neuroprotection in ERα activation induces phosphorylation of the protein kinase Akt [32]. To determine the efficiency of exogenous E_2_ treatment to promote intracellular activation of ERα signaling in our model, we evaluated the level of Akt phosphorylation (pAkt) in striatal protein samples (Fig 2C). The ratio pAkt / Akt increased with high E_2_, although this was not significantly different (p = 0.053 between O and E1, n = 6 per group).

To examine the relationship between circulating E_2_ and striatal GDNF, we performed a series of assays to monitor the expression of GDNF at both mRNA and protein levels. First, we tested the specificity of the quantitative RT-PCR using commercial TaqMan probes that amplify a cDNA sequence spanning exons 1 and exons 2. *Gdnf*^*+/-*^ mice carrying a single copy of the functional allele are expected to express about half of the normal GDNF levels. Indeed, *Gdnf*^*+/-*^ mice (n = 6) showed a significantly reduced striatal *Gdnf* mRNA content (normalized to *bActin* gene expression), being 42% of the *Gdnf* expression measured in *Gdnf*^*+/+*^ striatum (n = 9) (Fig 2D). ELISA was performed to assess the level of GDNF protein in the striatum. Since the adult mouse cortex does not express *Gdnf* (Fig C in S1 File), cortical proteins were run along with striatal proteins to set the background noise from unspecific binding (Fig C in S1 File). We found that the level of GDNF protein calculated were much lower in *Gdnf*^*+/-*^ mice (73 ± 26 pg/mg total protein) than in *Gdnf*^*+/+*^ mice (215 ± 25 pg/mg) (Fig 2E). A significant Pearson’s correlation (ρ = 0.72, p = 0.002) was found between *Gdnf* mRNA and GDNF protein levels (Fig 2F), which confirms the accuracy of the ELISA method employed in this study to specifically measure GDNF protein. In the ovx mice, no differences in *Gdnf* mRNA expression were observed among the four experimental groups (n = 5–6 / group) normalized with either *Parv* or *Hmbs* gene expression (Fig 2G). Moreover, we observed no variation either in GDNF protein content among the different E_2_ treatments (n = 6 / group) (Fig 2H). GDNF expression was assessed in two additional separate experiments, and we have never observed any increase of the GDNF levels in high E_2_ conditions (Fig D in S1 File).

**Fig 3:**
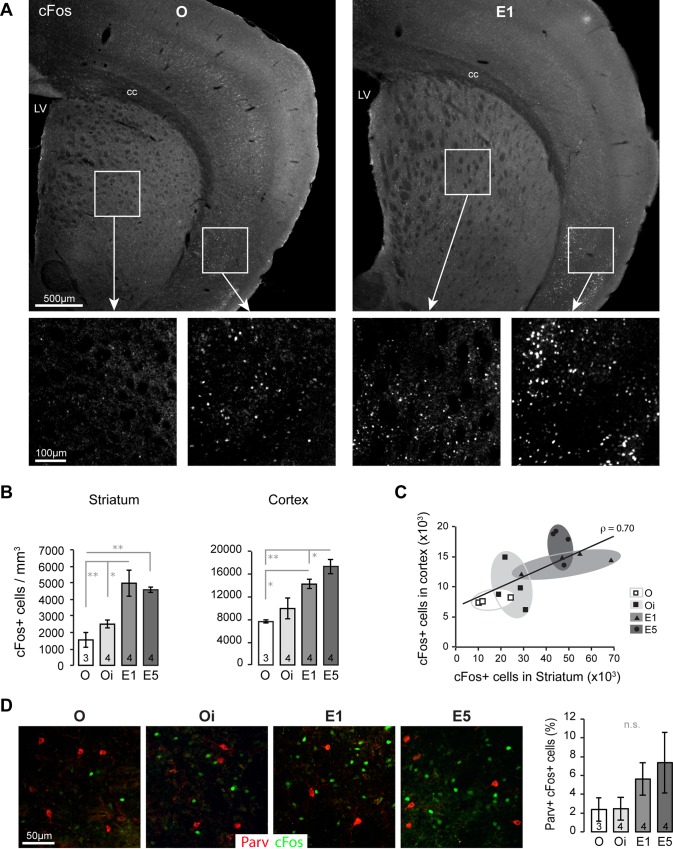
Induction of cFos by E_2_ in striatum and cortex. (A) Low magnification photomicrographs of O and E1 female mouse coronal sections illustrate the increase of cFos+ nuclei in the brain under E_2_ stimulation. Magnified views of striatum and cortex highlight the cFos staining difference between O and E1 mice. (B) Graphs showing the mean ± SEM of cFos+ nuclei in the striatum (left) and cortex (right) from O, Oi, E1 and E5 mice. (C) Correlation chart showing the positive correlation (ρ = 0.70, p = 0.004) between striatal and cortical number of cFos+ nuclei (shown in B). Note the gradual increase of cFos+ cells such as O < Oi < E1 ≤ E5. (D) Dual labeling for cFos (green) and Parv (red) showing the above mentioned increase of cFos+ nuclei in the striatum. Very few Parv+ cells are positive for cFos as indicated by the percentage of Parv+ cFos+ cells in the graph. n.s., not significant. *, p < 0.05; **, p < 0.01. N is indicated at the bottom of each bar.

Estrogen activates the proto-oncogene cFos expression in neuronal cell types [33,34]. Immunostaining for cFos followed by unbiased quantification revealed a progressive increase of the number of cFos-positive cells in the striatum and cortex (where ERα is highly present) in parallel with the rising plasma levels of E_2_ as illustrated in Fig 3A. cFos immunostaining shows relatively low expression in the striatum as compared to neighbor brain regions like the isocortex or the thalamic area (unpublished observations). In mice from group O, the number of cFos cells was 1544 ± 458 per mm^3^ (n = 3). The E_2_ SILASTIC implant in Oi mice induced a slight increase of cFos-positive cells (2503 ± 240, n = 4). However, elevated circulating E_2_ levels significantly stimulate striatal cFos expression (4996 ± 823 and 5591 ± 157 / mm^3^ in E1 and E5 respectively, n = 4) (Fig 3B). Noticeably, E_2_ induces cortical cFos expression in a dose-dependent manner (Fig 3B): O, 7646 ± 265 (n = 3); Oi, 9952 ± 1806 (n = 4); E1, 14264 ± 776 (n = 4) and E5, 17368 ± 1273 cFos^+^ cells / mm^3^ (n = 4). In addition, there is a significant correlation between striatal and cortical cFos expression (ρ = 0.70, p = 0.004) (Fig 3C). Dual labeling for Parv and cFos in the striatum demonstrated that few Parv neurons express cFos after high E_2_ stimulation (Fig 3D). O mice had 2.4 ± 1.3%, Oi mice had 2.47 ± 1.22%, E1 had a 5.62 ± 1.72% and E5 had 7.37 ± 3.24% Parv neurons expressing cFos. Surprisingly, we did not see any activation of the ERK1/2 p42/p44 MAPK pathway, a known activator of cFos, in the striatum (Fig E in S1 File). Although there is a trend indicating that circulating E_2_ induces cFos expression in Parv neurons, in our experiment this did not reach significance (p = 0.33), probably owing to the low number of cFos-positive Parv cells that generate high variability between individuals. The low response of Parv interneurons to high circulating E_2_, indicated by cFos induction, might help to explain why GDNF level does not vary in our animal model.

**Fig 4:**
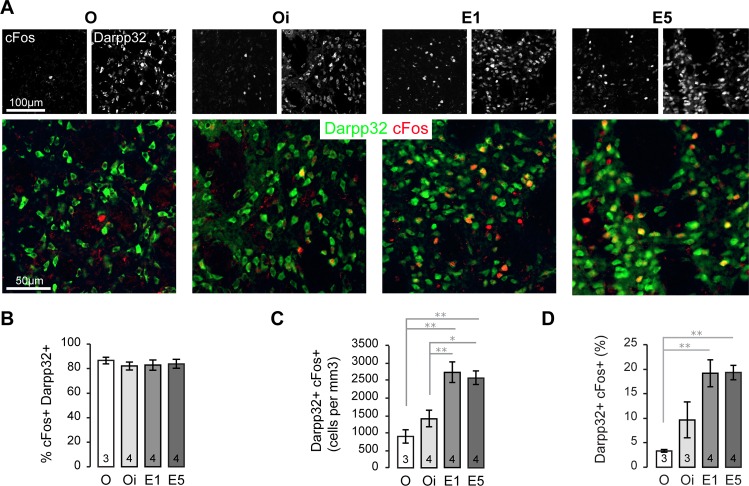
Induction of cFos by E_2_ in medium spiny neurons (MSN). (A) Photomicrographs of the dorsal striatum showing progressive increase of cFos+ (red) expression in Darpp32+ (green) MSN in O, Oi, E1 and E5 groups. (B) Graph showing the percentage of cFos+ nuclei expressed by Darpp32+ MSNs. (C) Graph showing the mean number ± SEM of Darpp32+ cFos+ cells. (D) Graph showing the mean percentage ± SEM of Darpp32+ that are cFos+. All data are presented as the mean ± SEM. *, p < 0.05; **, p < 0.01. N is indicated at the bottom of each bar.

Then, we looked for the cell type expressing cFos in response to high E_2_ and we found that medium spiny neurons (MSN) are the main cells activated by E_2_ in the striatum (Fig 4A). About 80% of cFos+ cells are Darpp32+ regardless of the E_2_ treatment (Fig 4B). Along with the general increase of cFos in the striatum, the number of Darpp32+ cells showing cFos induction significantly increases with rising E_2_: in O mice, 900 ± 193; in Oi mice, 1406 ± 231; in E1 mice, 2752 ± 306; and in E5 mice, 2573 ± 203 Darpp32+ cFos+ cells / mm^3^ (Fig 4C). Consequently, the percentage of Darpp32+ with cFos+ induction increase with E_2_: O, 3.3 ± 0.3%; Oi, 9.6 ± 3.6%; E1, 19.2 ± 2.8%; E5, 19.3 ± 1.5% (Fig 4D).

## Discussion

To further study the mechanism involved in the estrogen-mediated neuroprotection of the dopaminergic nigrostriatal pathway, we have evaluated the effect of plasma estrogen on GDNF production in the striatum using a model of ovariectomized (ovx) female mice. We found that virtually every parvalbumin (Parv) interneuron, the cell type producing most of the striatal GDNF [29], expresses the estrogen receptor alpha (ERα), with no sex dimorphism. Surprisingly, high E_2_ failed to induce GDNF production in ovx mice, and very few Parv interneurons displayed evidence of activation by E_2_, as evidenced by cFos induction. On the other hand, E_2_-induced cFos expression was broadly observed in the cortex and the striatum, mainly in medium spiny neurons (MSNs), suggesting a possible role for estrogen in the modulation of basal ganglia circuitry.

We report here the absence of GDNF increase under E_2_ exposure despite a general activation of ER intracellular pathways in the female adult mouse. These results differ from those obtained in other models, which varied in both experimental design and animal or cell types [25,26]. We used a model of ovx female mouse supplemented with exogenous E_2_, while others have used 6-OHDA-treated male rats [25] and ventral midbrain primary cultures [26] to show an increase of GDNF protein content by E_2_ stimulation. The methods to assess GDNF levels also vary between the studies. While other investigators privileged the use of polyclonal antibodies to detect GDNF by western blot [25,26], we favored quantitative RT-PCR to assay *Gdnf* gene expression, and ELISA to assess GDNF protein levels. In our study, slight variations exist between the different cohorts analyzed, and a decrease of striatal GDNF concentration has been occasionally observed upon high E_2_. The hormonal regimen used here has proven efficiency based on physiological changes such as vaginal cytology, Akt phosphorylation and cFos induction. Indeed, a 24 hours E_2_ exposure is enough to induce maximal neuronal activation (cFos) in the striatum. In spite of this, a 15-fold increase of plasma E_2_ over the baseline repeatedly failed in stimulating GDNF production. A speculative explanation to the lack of action of E_2_ on GDNF may reside in the absence of intracellular relay to the ERα in the Parv neurons. While ERα is likely to drive the E_2_ signal leading to neuroprotection by binding to specific response elements [16], a non-genomic action mediated by the G protein-coupled estrogen receptor (GPER) may occur as well. GPER mediates rapid estrogen signaling via neuronal extracellular signal-regulated kinases (ERKs) and phosphatidylinositol-3-kinase (PI3K)-Akt pathways [1,35], which promote neuron survival [36]. Nonetheless, a recent study reported no significant change in striatal GDNF levels in MPTP-treated male mice that were stimulated by E_2_, ERα, ERβ, and GPER agonists [35]. Also, the absence of ERα signaling lead to lower content in tyrosine hydroxylase and brain-derived neurotrophic factor (BDNF) in the midbrain of ERαKO mice, whereas GDNF remained unaffected [37]. Although obtained from mouse midbrain samples and not striatum, these data suggest that ERα activity may not be linked to GDNF production. With this in mind, the E_2_–mediated neuroprotection may occur through different pro-survival pathways (see for review [1]), as well as other local trophic factor synthesis, such as BDNF [37,38].

Since Parv interneurons participate to over 90% of the striatal GDNF production [24,29], it may be valuable to study the E_2_ neuroprotective effect in mice with conditional ERα deletion in the Parv neurons (*Parv-Cre / Esr1-flox* mouse model). Such model may help to clarify the divergence existing on the interaction between estrogen and GDNF in the neuroprotection of the nigrostriatal DA pathway.

Estrogen receptors (ERs) are expressed throughout the brain and both classical nuclear ERs α and β are found in the striatum [39], although ERβ shows a more restricted topographical expression than ERα [13]. Here we found that ERα is expressed in virtually every single projecting neuron (MSN) in the mouse striatum. High circulating E_2_ promotes cFos induction in various brain regions, but merely ~ 20% of the MSNs respond to exogenous E_2_. The sole presence of ERα may not be enough to activate every MSN, thus suggesting heterogeneity in the MSN population whereby some neurons are more sensitive to E_2_ than others. As yet, the effects of E_2_ on MSN are not well characterized. Estrogen can rapidly induce cFos expression via activation of both phosphatidylinositol 3-kinase/Akt and ERK1/2 mitogen-activated protein kinase [40,41]. However, we found no activation of the ERK1/2 pathway by ectopic E_2_ in the striatum. Instead, estrogen may trigger glutamatergic neuron activity in the cortex, since cFos induction is turned on by E_2_, and therefore indirectly modulate striatal MSN activity. Likewise, E_2_ is able to influence electrophysiological properties and dendritic plasticity of MSN [42], thus tuning the response of MSN to cortical glutamatergic inputs. Indeed, ERs are paired with metabotropic glutamate receptors (mGluRs) to mediate intracellular signaling such as mitogen-activated protein kinase (MAPK)-dependent CREB phosphorylation leading to transcription regulation in MSN [43]. Also, E_2_ is required for activity-dependent long-term potentiation in MSN [44], and it may as well affect the basal ganglia indirect pathway by modulating the D2-type DA receptor expression, such as in the rat dorsal striatum [45]. Moreover, E_2_ activates membrane-localized ERs to modulate DA synaptic neurotransmission [46], and potentiates the amphetamine-induced rotational behavior [47]. It is therefore possible that DA and E_2_ work together to activate a common cascade of intracellular events such as the cAMP-PKA and ERK pathways that are crucial for LTP. Interestingly, a neuroprotective effect of E_2_ has been reported in Huntington disease rat model, whereby females have a less severe course of the disease associated to higher resistance of MSNs [48]. In a behavioral perspective, estrogenic modulation of the DA and MSN function may underlie sex differences in motor and sensory functions, and for instance contribute to women’s ability to outperform men at tests of fine motor control and speech articulation [49].

In summary, estradiol treatment to ovariectomized mice was found to activate cFos expression in striatal MSNs, while there was little effect on the GDNF-producing parvalbumin interneurons. As a possible consequence to this, circulating E_2_ cannot stimulate GDNF production in this animal model. However, our results reinforce a role for estrogen treatment on the activation of a restricted MSN subpopulation that calls for further investigation.

## Supporting Information

S1 File **Fig A. Estrogen receptor alpha (ERα) expression in the female mouse striatum.** (A) Fluorescence microphotographs showing the broad ERα immunostaining in the striatum, motor and piriform cortex. (B) Confocal microphotograph of a single 1 micron plane showing ERα staining in parvalbumin (Parv) and medium spiny neurons (Darpp32). Note that ERα is expressed almost exclusively in Darpp32^+^ cells. (C) ERα expression (*Esr1* relative to *Hmbs*) does not vary between the different E_2_ treatments. N is indicated at the bottom of each bar. **Fig B. Vaginal smears representative cytology from mice in groups O, Oi, E1 and E5.** C, cornified cell typical of estrus stage when circulating sex steroids level is high; E, nucleated epithelial cell; L, leucocyte (neutrophil). **Fig C. GDNF ELISA specificity.** Since adult mouse cortex does not express *Gdnf*, cortex proteins are used to set the non-specific binding in GDNF ELISA experiment. (A) QPCR analysis of *Gdnf1-2* (relative to *Actb*) in cortex and striatum of the adult female mouse (n = 4). (B) Scattered plot showing raw absorbance (optical density at 450nm) measured in the female mouse cortex (n = 4) and in the striatum of mice from O, Oi, E1, and E5 groups (n = 6). **Fig D. Analysis of GDNF levels in the O, Oi, E1 and E5 mice in two separate experiments (A-C and D-H).** (A) QPCR analysis of *Gdnf1-2* relative to *Hmbs* in striatum and cortex. (B) QPCR analysis of *Gdnf2-3* relative to *Hmbs* in striatum and cortex. (C) QPCR analysis of *Pvalb* relative to *Hmbs* in striatum and cortex. (D, E) QPCR analysis of *Gdnf1-2* (D) and *Gdnf2-3* (E) relative to *Hmbs* in the striatum. (F, G) QPCR analysis of *Gdnf1-2* (F) and *Gdnf2-3* (G) relative to *Actb* in the striatum. (H) GDNF protein levels in the striatum. n.s., non significant difference. N is indicated at the bottom of each bar. **Fig E. Phosphorylation of Erk1/2 (pErk1/2) in the mouse striatum.** Western blot analysis of striatal proteins shows no difference between ovx (O), ovx + E_2_ implant (Oi), ovx + E_2_ implant + E_2_ s.c 1 day (E1) and ovx + E_2_ implant + E_2_ s.c 5 days (E5). *Left*, representative blot. *Right*, bar graph illustrating the pErk1/2 / Erk1/2 ratio. n.s., non significant difference. N is indicated at the bottom of each bar.
(PDF)
