# Supplementary material for: Striatal GDNF Production Is Independent to Circulating Estradiol Level Despite Pan-Neuronal Activation in the Female Mouse
Source: PLoS One. 2016 Oct 14;11(10):e0164391. doi: 10.1371/journal.pone.0164391 (PMC5065215; doi:10.1371/journal.pone.0164391)
Supplement: S1 File — Fig A. Estrogen receptor alpha (ERα) expression in the female mouse striatum. (A) Fluorescence microphotographs showing the broad ERα immunostaining in the striatum, motor and piriform cortex. (B) Confocal microphotograph of a single 1 micron plane showing ERα staining in parvalbumin (Parv) and medium spiny neurons (Darpp32). Note that ERα is expressed almost exclusively in Darpp32+ cells. (C) ERα expression (Esr1 relative to Hmbs) does not vary between the different E2 treatments. N is indicated at the bottom of each bar. Fig B. Vaginal smears representative cytology from mice in groups O, Oi, E1 and E5. C, cornified cell typical of estrus stage when circulating sex steroids level is high; E, nucleated epithelial cell; L, leucocyte (neutrophil). Fig C. GDNF ELISA specificity. Since adult mouse cortex does not express Gdnf, cortex proteins are used to set the non-specific binding in GDNF ELISA experiment. (A) QPCR analysis of Gdnf1-2 (relative to Actb) in cortex and striatum of the adult female mouse (n = 4). (B) Scattered plot showing raw absorbance (optical density at 450nm) measured in the female mouse cortex (n = 4) and in the striatum of mice from O, Oi, E1, and E5 groups (n = 6). Fig D. Analysis of GDNF levels in the O, Oi, E1 and E5 mice in two separate experiments (A-C and D-H). (A) QPCR analysis of Gdnf1-2 relative to Hmbs in striatum and cortex. (B) QPCR analysis of Gdnf2-3 relative to Hmbs in striatum and cortex. (C) QPCR analysis of Pvalb relative to Hmbs in striatum and cortex. (D, E) QPCR analysis of Gdnf1-2 (D) and Gdnf2-3 (E) relative to Hmbs in the striatum. (F, G) QPCR analysis of Gdnf1-2 (F) and Gdnf2-3 (G) relative to Actb in the striatum. (H) GDNF protein levels in the striatum. n.s., non significant difference. N is indicated at the bottom of each bar. Fig E. Phosphorylation of Erk1/2 (pErk1/2) in the mouse striatum. Western blot analysis of striatal proteins shows no difference between ovx (O), ovx + E2 implant (Oi), ovx + E2 implant + E2 s.c [file pone.0164391.s001.pdf]

Supporting Information

Supplementary Methods

Vaginal smear samples

Vaginal lavage was performed to anesthetized mice by using a plastic pipette filled with saline solution. A small drop of the sample was placed evenly on a slide in a thin layer (smear) and allowed to air dry. Slides were stained with methylene blue 10% v/v (03978, Sigma) and evaluated under microscope (as recommended in Cora et al. Toxicol Pathol. 2015;43:776–93).

Real-time quantitative PCR (QPCR)

The TaqMan® probes (Thermofisher) used are:

Esr1, Mm00433149_m1.

Gdnf2-3, Fwd: GTGACTCCAATATGCCTGAAGATTATC,
Rev: TCAGTCTTTTAATGGTGGCTTGAA,
Internal probe: TGACCAGTTTGATGACGTC.

Immunohistochemistry

Triple immunostaining for Parv, ERα and Darpp32 using antibody dilutions was performed as described in the main article. An anti-goat IgG Alexa® Fluor 633 was used for Parv antibody detection. 1micron slice images in the z-plan were obtained with a Leica TCS SP2 confocal microscope.

Western blot

Protein quantity, protocol steps and quantification of Erk1/2 phosphorylation were performed as described in the main article. Anti-Phospho-p44/42 MAPK (pErk1/2) (Thr202/Tyr204) (9101, Cell Signaling) and anti-p44/42 MAPK (Erk1/2) antibodies were used at 1:1000 dilution.

Supplementary Figures

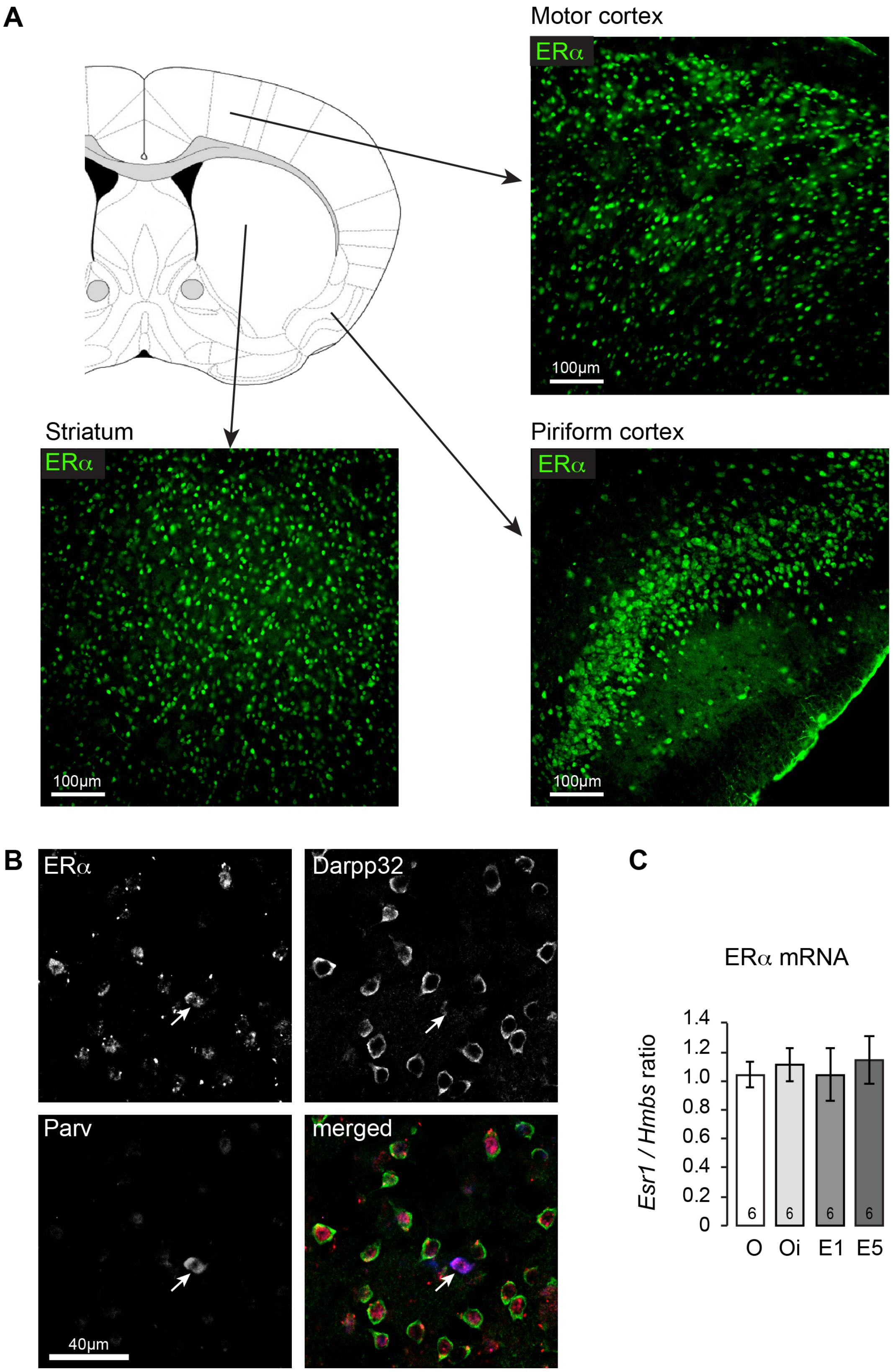

Fig A. Estrogen receptor alpha (ERα) expression in the female mouse striatum.

(A) Fluorescence microphotographs showing the broad ERα immunostaining in the striatum, motor and piriform cortex. (B) Confocal microphotograph of a single 1 micron plane showing ERα staining in parvalbumin (Parv) and medium spiny neurons (Darpp32). Note that ERα is expressed almost exclusively in Darpp32^{+} cells. (C) ERα expression (*Esr1* relative to *Hmbs*) does not vary between the different E_2 treatments. N is indicated at the bottom of each bar.

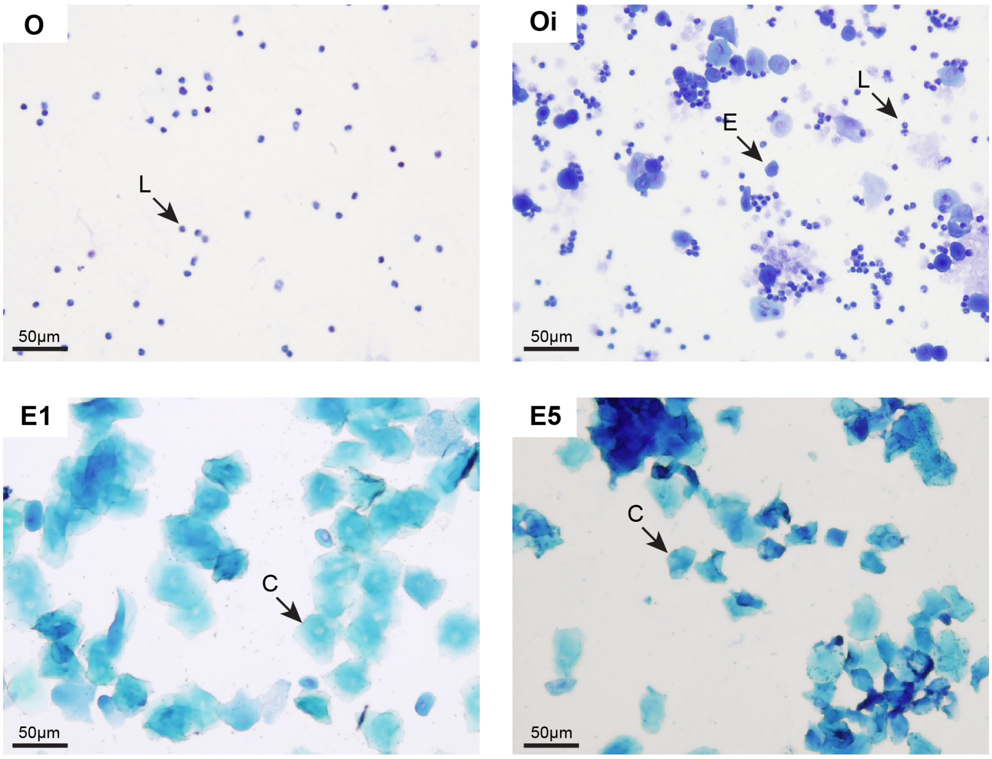

Fig B. Vaginal smears representative cytology from mice in groups O, Oi, E1 and E5.

C, cornified cell typical of estrus stage when circulating sex steroids level is high; E, nucleated epithelial cell; L, leucocyte (neutrophil).

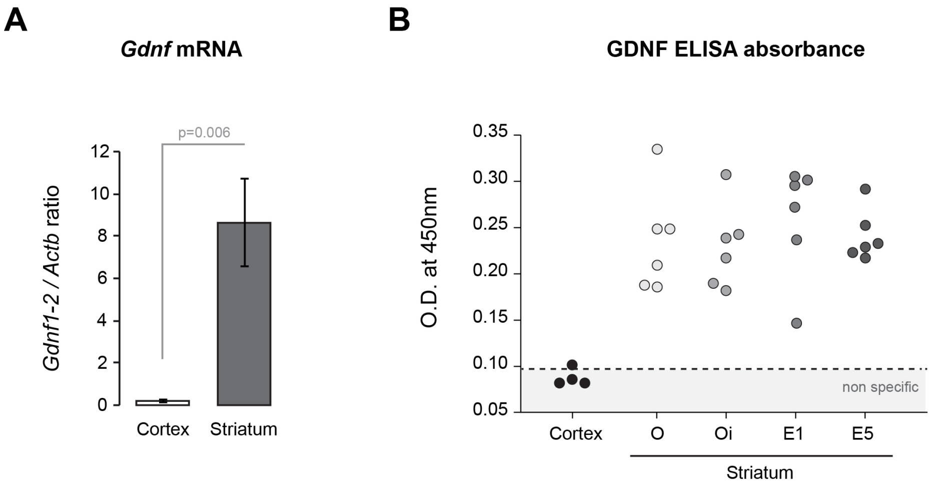

Fig C. GDNF ELISA specificity.

Since adult mouse cortex does not express *Gdnf*, cortex proteins are used to set the non-specific binding in GDNF ELISA experiment. (A) QPCR analysis of *Gdnf1-2* (relative to *Actb*) in cortex and striatum of the adult female mouse (n = 4). (B) Scattered plot showing raw absorbance (optical density at 450nm) measured in the female mouse cortex (n = 4) and in the striatum of mice from O, Oi, E1, and E5 groups (n = 6).

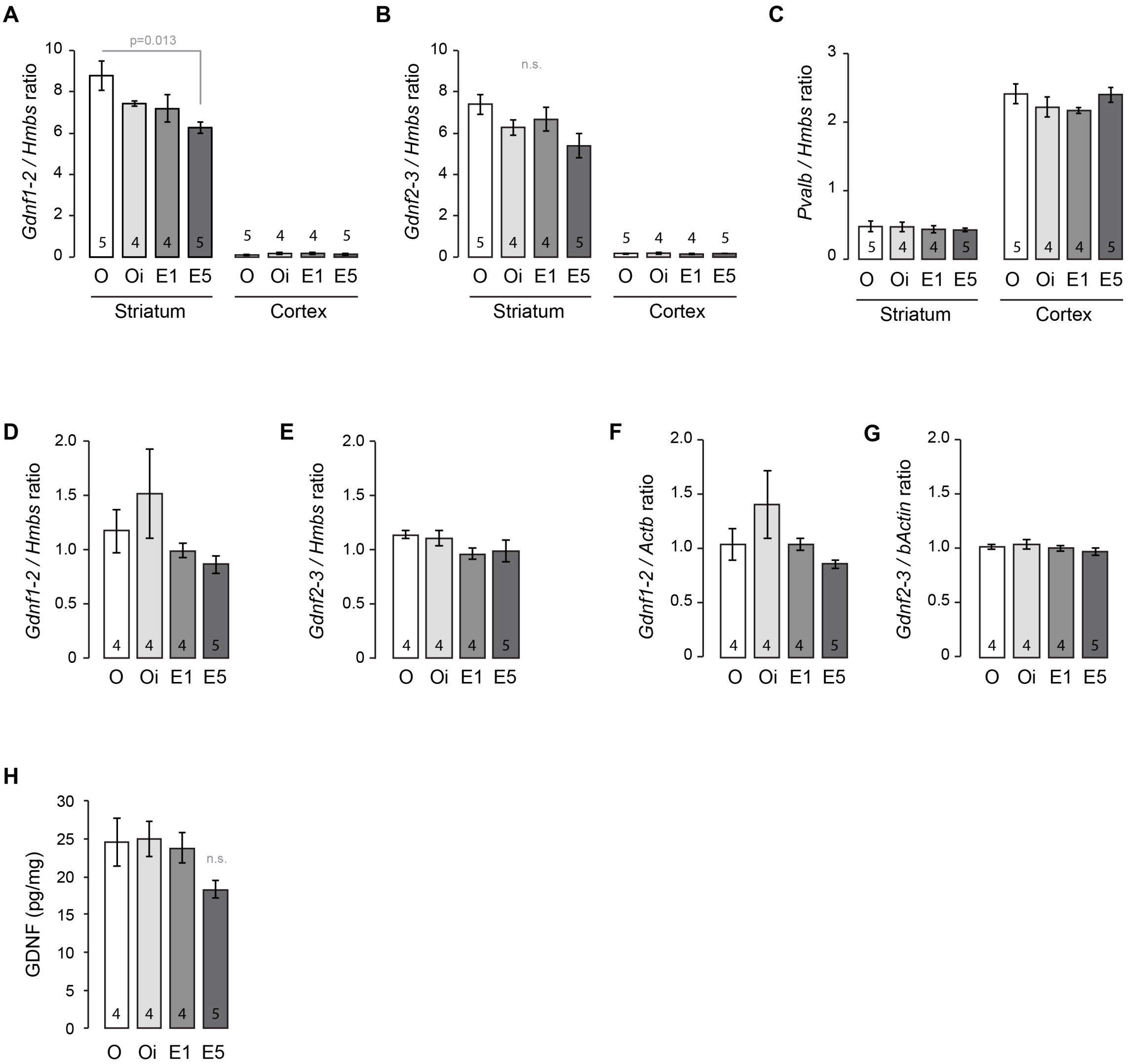

Fig D. Analysis of GDNF levels in the O, Oi, E1 and E5 mice in two separate experiments (A-C and D-H).

(A) QPCR analysis of *Gdnf1-2* relative to *Hmbs* in striatum and cortex. (B) QPCR analysis of *Gdnf2-3* relative to *Hmbs* in striatum and cortex. (C) QPCR analysis of *Pvalb* relative to *Hmbs* in striatum and cortex. (D, E) QPCR analysis of *Gdnf1-2* (D) and *Gdnf2-3* (E) relative to *Hmbs* in the striatum. (F, G) QPCR analysis of *Gdnf1-2* (F) and *Gdnf2-3* (G) relative to *Actb* in the striatum. (H) GDNF protein levels in the striatum. n.s., non significant difference. N is indicated at the bottom of each bar.

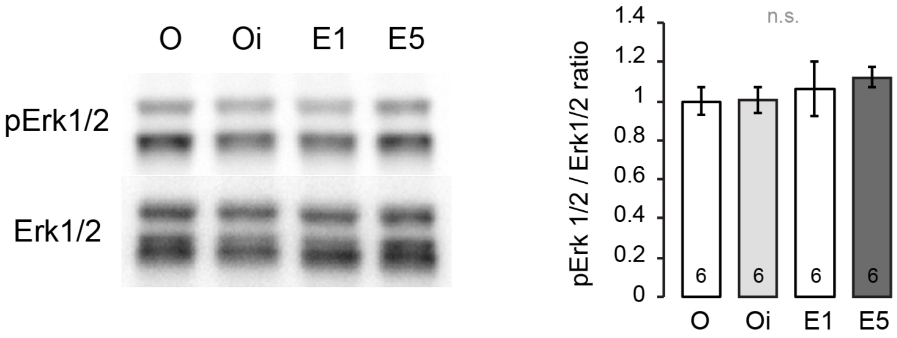

Fig E. Phosphorylation of Erk1/2 (pErk1/2) in the mouse striatum.

Western blot analysis of striatal proteins shows no difference between ovx (O), ovx + E_2 implant (Oi), ovx + E_2 implant + E_2 s.c 1 day (E1) and ovx + E_2 implant + E_2 s.c 5 days (E5). *Left*, representative blot. *Right*, bar graph illustrating the pErk1/2 / Erk1/2 ratio. n.s., non significant difference. N is indicated at the bottom of each bar.
